# A Clinical Radiomics Nomogram Was Developed by Integrating Radiomics Signatures and Clinical Variables to Distinguish High-Grade ccRCC from Type 2 pRCC

**DOI:** 10.1155/2022/6844349

**Published:** 2022-08-26

**Authors:** Yankun Gao, Xiaoying Zhao, Xia Wang, Chao Zhu, Cuiping Li, Jianying Li, Xingwang Wu

**Affiliations:** ^1^Department of Radiology, The First Affiliated Hospital of Anhui Medical University, Hefei 230022, Anhui, China; ^2^CT Research Center, GE Healthcare China, Shanghai 210000, China

## Abstract

**Purpose:**

A nomogram was constructed by combining clinical factors and a CT-based radiomics signature to discriminate between high-grade clear cell renal cell carcinoma (ccRCC) and type 2 papillary renal cell carcinoma (pRCC).

**Methods:**

A total of 142 patients with 71 in high-grade ccRCC and seventy-one in type 2 pRCC were enrolled and split into a training cohort (*n* = 98) and a testing cohort (*n* = 44). A clinical factor model containing patient demographics and CT imaging characteristics was designed. By extracting the radiomics features from the precontrast phase, corticomedullary phase (CMP), and nephrographic phase (NP) CT images, a radiomics signature was established, and a Rad-score was computed. By combining the Rad-score and significant clinical factors using multivariate logistic regression analysis, a clinical radiomics nomogram was subsequently developed. The diagnostic performance of these three models was evaluated by using data from both the training and testing groups using a receiver operating characteristic (ROC) curve analysis.

**Results:**

The radiomics signature contained eight validated features from the CT images. The relative enhancement value of CMP (REV1) was an independent risk factor in the clinical factor model. The area under the curve (AUC) value of the clinical radiomics nomogram was 0.974 and 0.952 in the training and testing cohorts, respectively. In the training cohort, the decision curves of the nomogram demonstrated an added overall net advantage compared to the clinical factor model.

**Conclusion:**

A noninvasive prediction tool termed radiomics nomogram, combining clinical criteria and the radiomics signature, may accurately predict high-grade ccRCC and type 2 pRCC before surgery. It also has some importance in assisting clinicians in determining future treatment strategies.

## 1. Introduction

Approximately, 85% of renal tumors in adults are in the form of renal cell carcinoma (RCC) [1]. Moreover, 70-80%, 10-20%, and 3-7% of RCCs are clear cell RCC (ccRCC), papillary RCC (pRCC), and chromophobe RCC (chRCC), respectively [2]. The ccRCC is the most frequent subtype of RCC, which is prone to vascular invasion and early metastases. As a result, early detection and treatment are critical. According to the Fuhrman nuclear grading system, the ccRCC is categorized into four grades (i.e., I, II, III, and IV) [3]. Compared to grades III and IV ccRCC, grades I and II ccRCC are low-grade tumors with better prognoses. The incidence of pRCC is second only to ccRCC, and pRCC was initially divided into types 1 and 2 based on morphological and immunohistochemical characteristics by Delahunt et al. [4]. Previous research has shown that type 2 pRCC has a higher pathological stage, higher nuclear grade, and poor prognosis [5, 6]. There are significant differences in prognosis between ccRCC and pRCC. The ccRCC has a poor prognosis, and the 5-year survival rate is significantly lower than pRCC [7, 8].

Surgical excision is the primary treatment for renal cell carcinoma (RCC). However, the surgical method varies according to the RCC subtype. The ccRCC typically necessitates surgical removal of the entire kidney, whereas pRCC allows preservation of the nephron [9–11]. In addition, new targeted therapies and drugs are being used in clinical care [12, 13]. However, ccRCC and pRCC respond differently to targeted therapy, especially for high-grade ccRCC and type 2 pRCC. High-grade ccRCC and type 2 pRCC are advanced RCCs that are often lost to surgery and can only be treated with medication. However, different drugs are used for the two subtypes of RCC, so accurately identifying the tumor subtype before drug administration will help develop a treatment strategy.

Pathological biopsy by percutaneous puncture is an excellent way to differentiate between high-grade ccRCC and type 2 pRCC but it is invasive and most commonly causes bleeding. Many studies have recently focused on this problem. Young et al. [8] have proposed that multiphasic MDCT may help differentiate ccRCC from oncocytoma, pRCC, and chRCC. Similarly, Sun et al. [14] demonstrated that studies using dynamic contrast-enhanced MRI, ccRCC, and non-ccRCC had different enhancement patterns. Liang et al. [15] energy spectrum CT parameters could identify high-grade ccRCC and type 2 pRCC. Nevertheless, on regular CT or MRI images, these two tumor types have similar imaging features. By imaging features alone, it is challenging to distinguish high-grade ccRCC from type 2 pRCC.

In recent years, advances in radiomics have improved the prediction and categorization of cancer, particularly kidney tumors. Deng et al. [16] found that CT texture analysis helped distinguish pRCC from ccRCC and predicted the Fuhrman grading. Wang et al. [11] showed that radiomics features could be extracted from multisequence MRI to differentiate between ccRCC, pRCC, and chRCC. Ma et al. [17] also found that a radiomics nomogram based on enhanced CT images could differentiate renal angiomyolipoma with minimal fat from ccRCC. Only a few studies have used CT-based radiomics nomograms to distinguish type 2 pRCC from high-grade ccRCC. In the present study, we quantitatively evaluated radiomics signatures using the Rad-score value of each patient and established a CT-based radiomics nomogram for differentiating type 2 pRCC from high-grade ccRCC by integrating both the Rad-score and conventional imaging features.

## 2. Materials and Methods

### 2.1. Patients

The retrospective study was approved by the ethics review board of our hospital, and patient informed consent was waived due to the retrospective nature of this study. Patients who underwent both precontrast and contrast-enhanced CT scans between February 2013 and November 2021 for RCC diagnosis at The First Affiliated Hospital of Anhui Medical University were considered. The inclusion criteria included the following: (1) all patients who were diagnosed with high-grade ccRCC or type 2 pRCC by pathology; (2) patients who had a preoperative unenhanced and contrast-enhanced CT examination with diagnostic image quality; and (3) patients who had complete clinicopathological data. The exclusion criteria included the following: (1) patients with a pathological diagnosis of low-grade ccRCC or type 1 pRCC; (2) patients who had other tumors; and (3) patients who had undergone radiotherapy or chemotherapy before CT examination.

### 2.2. Image Acquisition on CT

All patients underwent CT examination, including a precontrast phase, corticomedullary phase (CMP, obtained 30 s after contrast injection), nephrographic phase (NP, obtained 80 s after contrast injection), and excretory phase (EP, obtained 180 s after contrast injection). The contrast agent (320 mg/mL, Omnipaque, GE Healthcare) was injected via peripheral veins at a dose of 1.5 mL/kg of body weight and a flow rate of 3.0 mL/s.  Table 1 displays the CT scan parameters utilized in this study.

### 2.3. Evaluation of CT Scan Characteristics

Two radiologists (reader 1, Y.G. with 5 years of experience in abdominal CT diagnosis; reader 2, X.W. with eight years of experience in abdominal CT diagnosis) reviewed the axial CT images independently without prior knowledge of the patient's clinicopathological findings. These include the following characteristics of the tumors: maximum diameter, shape (round or not round), location (left or right), boundary (well defined or blurred), calcification (present or not present, defined as “areas with high CT values in non-contrast enhanced CT”), necrosis (present or not present, defined as “the nonenhanced area in the tumor that is more than 50% of the tumor”), renal vein invasion (present or not present, defined as “the tumor tissue observed in the renal vein and inferior vena cava”), and lymph node metastasis (present or not present, defined as “the perirenal and retroperitoneal lymph nodes with the short-axis diameter greater than 10 mm”) [18]. Final decision was reached by consensus if there was disagreement between the two radiologists.

**Figure 1:**
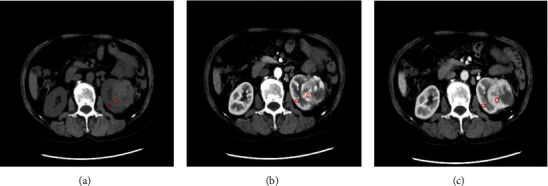
Example of selection the region of interest (ROI) for tumor and reference region. (a), (b), and (c) correspond to the image in the precontrast phase, corticomedullary phase (CMP), and nephrographic phase (NP), respectively. The red circle is for the tumor in three phases. The red oval represents the region in the kidney cortical section used as the reference. These ROIs were consistent for the three scan phases.

Tumor enhancement was measured in different scan phases by selecting the appropriate region of interest (ROI) within the tumor. Since tumors are enhanced and their heterogeneous components are evident in CMP, all ROIs were first selected using the CMP images. The ROIs included only the notable features of the tumors, avoiding elements such as necrosis, calcification, and visible vascularity in the images. Reader 1 performed three nonoverlapping ROIs, separate measurements using three different ROIs on the tumor, and obtained the final value by averaging the three measurements. To reduce the enhancement variation due to the blood circulation differences in individual patients and other factors related to scanning operations, similar measurements were obtained in the kidney cortical section on the same side of the tumor and were used as the references for normalizing the enhancement measurement. An illustration of this method is shown in Figure 1.

The ROIs selected in CMP were propagated into the precontrast phase and NP images to obtain an average tumor attenuation value (TAV) in each phase. The cortex attenuation value (CAV) was calculated using the average CT value of the reference area for each corresponding scan phase. By subtracting the values of the same ROI in the precontrast phase, the tumor enhancement value (TEV) and the cortex enhancement value (CEV) were calculated: TEV_x_ = TAV_x_−TAV_0_ and CEV_x_ = CAV_x_−CAV_0_, where *x* stands for the phase (0, precontrast phase; 1, CMP; 2, NP). The ratio of TEV to CEV was used to define the relative enhancement value (REV) : REV_x_ = TEV_x_/CEV_x_, which represents the degree of enhancement within the tumor relative to the renal cortex [19].

### 2.4. Construction of the Clinical Factor Model

The differences in clinical factors, which include clinical data and CT features, between the high-grade ccRCC and type 2 pRCC were compared using univariate analysis. A multivariate logistic regression analysis was then applied to establish a clinical factor model using the significant variables obtained in the univariate analysis. For each independent factor, odds ratios (OR) were estimated as a relative risk estimate with a 95% confidence interval (CI).

### 2.5. Tumor Image Segmentation in Three-Dimensional and Feature Extraction from Radiomics

**Figure 2:**
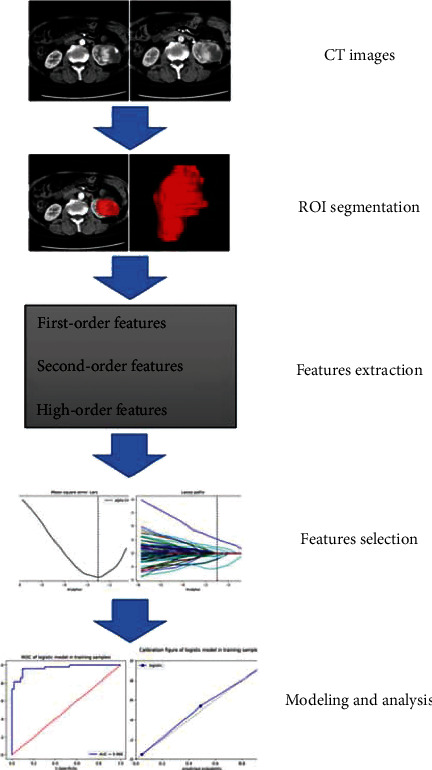
Schematic representation of a radiomics study of renal tumors.

Figure 2 shows the fundamental steps of a radiomics model for kidney tumors. The ITK-SNAP software (version 3.8, http://www.itksnap.org) was used to segment tumors in three-dimension (3-D). On the precontrast phase, CMP, and NP images, tumor borders were drawn using the ROI tool 1-2 mm away from the tumor border. Figure S1 displays an illustration of manual segmentation in a kidney tumor.

The PHIgo Workstation (General Electric Company, USA) was used to perform the feature extraction. Before features could be extracted from the ROIs of the three phase images, normalization and image resampling had to be carried out because the images were taken from three scanners with varied parameters. The image data are normalized using a z-score in the following formula:

(1) $$\begin{array}{c} z=\frac{x-\mu }{\sigma }, \end{array}$$

where *μ* is the mean of the entire set of data and *σ* is the standard deviation of the actual data. Furthermore, using B-spline interpolation sampling technology, all CT images were resampled to 1.0 × 1.0 × 1.0 mm^3^ voxels to standardize the slice thickness. The precontrast phase, CMP, and NP images, each contained 1595 radiomics features that were extracted.

Typically, inter- and intraclass correlation coefficients (ICC) were used to evaluate the radiomics feature extraction process' inter-observer reliability and intraobserver repeatability. The CT images of a total of 20 cases (10 high-grade ccRCC and ten types 2 pRCC) were randomly selected from the patient cohort for ROI segmentation by both readers. Reader 1 repeated the identical procedures two weeks later to assess the degree of feature extraction matching. The extracted features are considered to have good consistency when the ICC value is greater than 0.75 and the remaining image segmentation will be carried out by reader one alone.

### 2.6. Construction of the Radiomics Signature

Additional feature selection occurred before creating the radiomics signature to avoid overfitting. First, characteristics from the training cohort with an ICC >0.75 were preserved. Second, statistically insignificant factors were eliminated using univariate logistic analysis. The least absolute shrinkage and selection operator (LASSO) regression model was then utilized to identify the most valuable variables. A radiomics score (Rad-score) was finally derived based on radiomics properties to build the radiomics signature using multivariate logistic regression.

### 2.7. Construction of the Radiomics Nomogram and Performance Evaluation of Different Models

By merging the significant variables of clinical considerations and the Rad-score, a clinical radiomics nomogram was created. Calibration curves were utilized to assess the nomogram's calibration. The goodness-of-fit of the nomogram was assessed using the Hosmer–Lemeshow test. The area under the receiver operator characteristic (ROC) curve was used to assess the diagnostic performance of the clinical factors model, the radiomics signature model, and the clinical radiomics nomogram in differentiating high-grade ccRCC from type 2 pRCC in both the training and testing cohorts. A decision curve analysis (DCA) was performed to measure the clinical effectiveness of the radiomics nomogram by estimating the net benefit of a threshold possibility range throughout the training and testing groups.

### 2.8. Statistical Analysis

The SPSS (version 25.0, IBM) and IPM (version 2.4.0, General Electric Company) statistical packages were used for statistical analysis. A univariate analysis was performed to assess the differences in clinical variables between high-grade ccRCC and type 2 pRCC. For categorical variables, the Chi-square or Fisher exact tests were utilized. The one-sample Kolmogorov–Smirnov test was used to examine whether the numerical variables had a normal distribution. Normally, distributed data are expressed as the mean, standard deviation (*M* ± SD), and non-normally distributed data are defined as the median (IQR), 25th, and 75th percentiles. An independent sample *t *test was used for data conforming to a normal distribution.

In contrast, a Mann–Whitney *U* test (a nonparametric rank-sum test) was used for data conforming to a nonnormal distribution. The ROC curve analysis was performed to determine the area-under-the-curve (AUC), accuracy, specificity, and sensitivity to evaluate the model's performance. The Delong test model was used for the statistical comparison of ROC curves. A two-sided *p* < 0.05 was considered significant.

## 3. Results

### 3.1. Clinical Factors of the Patients and Construction of the Clinical Factor Model

Our study eventually enrolled 142 patients, including 71 high-grade ccRCC patients (54 males and 17 females) and 71 type 2 pRCC patients (55 males and 16 females). Patients were randomly divided into the training (*n* = 98) and testing (*n* = 44) cohorts in a 7 : 3 ratio.  Table 2 displays the clinical factors data from the training and testing cohorts. TEV1, TEV2, REV1, and REV2 were statistically significant in differentiating high-grade ccRCC and type 2 pRCC using the univariate analysis with data in the training group (both *p* < 0.001). These four statistically significant clinical factors identified above were then subjected to the multivariate logistic regression analyses. The *p* value were 0.179, 0.718, <0.001, and 0.812 using TEV1, TEV2, REV1, and REV2, respectively. REV1 acts as an independent predictor. A higher value (OR, 1.053; 95%CI; 1.031–1.077) indicates a higher likelihood of high-grade ccRCC.

### 3.2. Features Extraction, Selection, and Construction of Radiomics Signature

**Figure 3:**
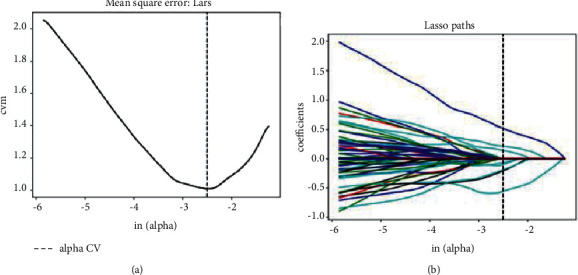
Radiomics features were selected using a minor absolute shrinkage and selection operator (LASSO) regression model. (a). The LASSO model's tuning parameter (*λ*) was determined using a minimum criterion 10-fold cross-validation criterion. The dotted vertical lines indicate the optimal values of the LASSO tuning parameter (*λ*), and a value *λ* of 0.082 with ln (*λ*) = −2.501 was chosen. (b). LASSO coefficient of the 427 radiomics features. A coefficient profile plot was generated versus the selected ln (*λ*) value using 10-fold cross-validation; the vertical line was plotted with eight chosen radiomics features.

From the precontrast phase, CMP, and NP CT images, a total of 4785 radiomics features were extracted, with 3678 of them having ICCs greater than 0.75, demonstrating good inter- and intraobserver consistency. Univariate correlation analysis revealed significant differences in 427 radiomic characteristics between high-grade ccRCC and type 2 pRCC. Eight significant features were obtained by successively importing these features into the LASSO logistic regression model to identify the most vital features (Figure 3). Finally, eight features were used to construct the radiomics signature. In the training group, the AUC was 0.968 (95% CI 0.941–0.990); in the testing cohort, it was 0.936 (95% CI 0.876–0.986). The Rad-score was computed using the formula given below:

(2) $$\begin{array}{c} \text{Rad}-\text{score }=\;-0.4531+\left(-0.8374\;\times \;P\_\text{original}\_\text{glszm}\_\text{SmallAreaLowGrayLeveIEmphasis}\right)\\ +\left(-0.6712\;\times \;P\_\text{wavelet}\_\text{HLH}\_\text{glcm}\_\text{ClusterShade}\right)\\ +\left(-1.1904\;\times \text{ CMP}\_\text{wavelet}\_\text{HLL}\_\text{glszm}\_\text{SizeZoneNonUniformityNormalized}\right)\\ +\left(-1.2775\;\times \text{ CMP\_wavelet\_LHH\_glcm\_ClusterShade}\right)\\ +\left(1.2992\;\times \text{ CMP\_wavelet\_LLL\_firstorder\_InterquartquartileRange}\right)\\ +\left(0.4960\;\times \text{ CMP\_wavelet\_LLL\_glcm\_InverseVariance}\right)\\ +\left(-0.8209\;\times \text{ NP\_log\_sigma}\_1\_0\text{mm\_}3\text{D\_glszm\_SizeZoneNonUniformityNormalized}\right)\\ +\left(0.7470\;\times \text{ NP\_wavelet\_LLL\_firstorder\_InterquartileRange}\right). \end{array}$$

### 3.3. Establishment of the Radiomics Nomogram and Performance Evaluation among Different Models

**Figure 4:**
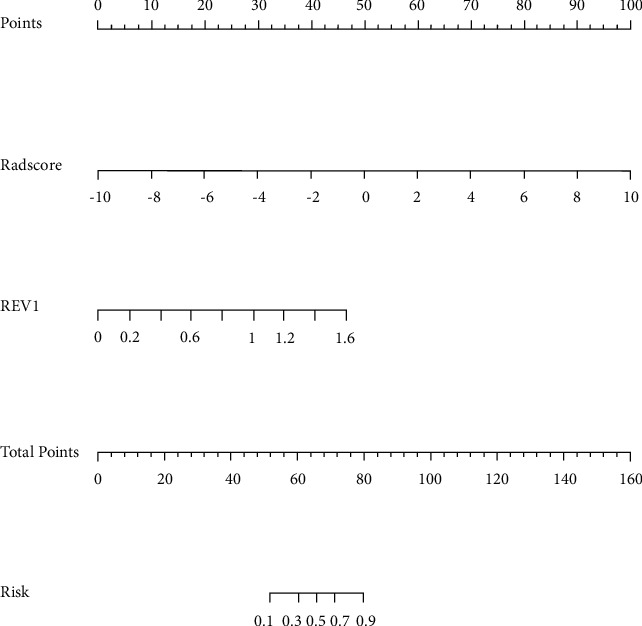
A radiomics nomogram distinguishing between high-grade clear cell renal cell carcinoma (ccRCC) and type 2 papillary renal cell carcinoma (pRCC). Based on a training cohort, the nomogram was created by combining the relative enhancement value of the corticomedullary phase (REV1) and radiomics score (Rad-score).

A clinical radiomics nomogram was developed by integrating the REV1 and the Rad-score, two essential clinical parameters using the data in the training cohort (Figure 4). Multivariate logistic regression was used to generate the radiomics nomogram score (Nomo-score). The formula for calculating the Nomo-score for this study is given below:

(3) $$\begin{array}{c} \text{Nomo}-\text{score}=\;-2.4533+\text{Rad}-\text{score}\times \;0.2284+\text{REV}1\times \;4.5795. \end{array}$$

**Figure 5:**
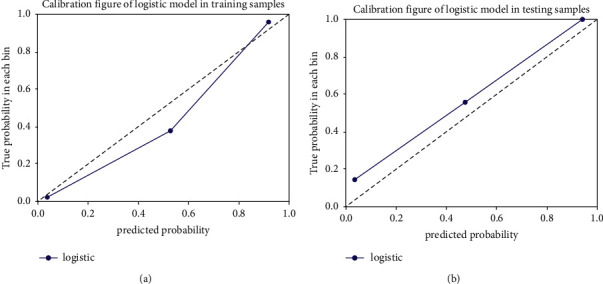
The calibration curves for the radiomics nomogram (solid lines) in the training (a) and testing (b) cohorts indicate a good fit for the radiomics nomogram. The closer the distance between the solid line and the 45° straight dash line, the better the accuracy of the calibration curve.

**Figure 6:**
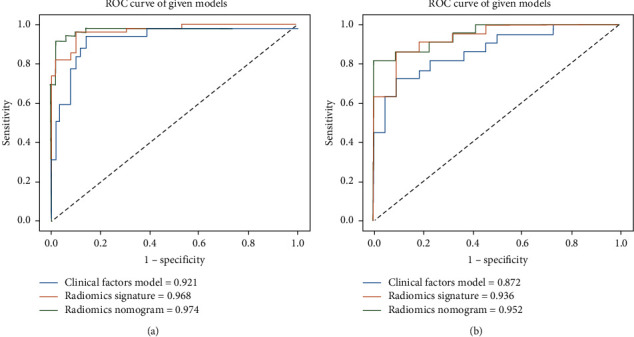
The radiomics nomogram, radiomics signature, and clinical factors model receiver operating characteristic (ROC) curves for training (a) and testing (b) cohorts.

**Figure 7:**
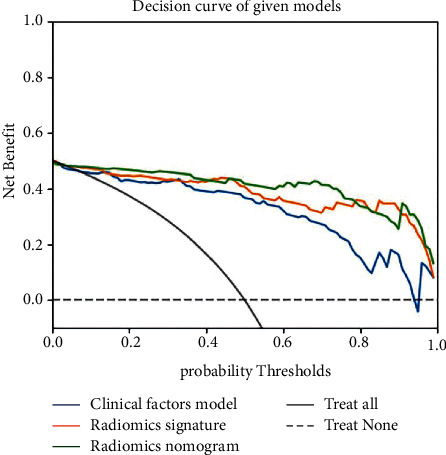
Decision curve analysis (DCA) for the clinical factors model (blue line), radiomics signature (yellow line), and clinical radiomics nomogram (green line). The *Y* axis indicates the net benefit; the *X* axis indicates probability thresholds. The clinical radiomics nomogram and radiomics signature outperformed the clinical factors model in distinguishing high-grade clear cell renal cell carcinoma (ccRCC) from type 2 papillary renal cell carcinoma (pRCC).

Figure 5 depicts the calibration curves for the clinical radiomics nomogram showing good calibration with data in both training and testing groups.  Table 3 displays the discriminatory efficiencies of the clinical factors model, radiomics signature, and clinical radiomics nomogram. In both the training and testing cohorts, the AUC values for the clinical radiomics nomogram were higher than those of the radiomics signature and the clinical factors model (*p*=0.062, 0.010; *p*=0.210, 0.070).  Figure 6 compares the accuracies of the three models in differentiating high-grade ccRCC from type 2 pRCC using the ROC curves based on the training and testing cohorts. The decision curves demonstrated that when compared to the clinical factors and radiomics signature, the clinical radiomics nomogram added a more significant overall benefit in distinguishing between high-grade ccRCC and type 2 pRCC in most of the training cohorts within reasonable threshold probabilities. The DCA values for the three models in the training cohort are shown in Figure 7.

## 4. Discussion

The two most frequent malignant renal tumors are ccRCC and pRCC. Earlier studies reported that type 2 pRCC is more malignant than type 1 pRCC and that type 2 pRCC is more severe [20]. Zhu et al. [19] showed that the enhancement of high-grade ccRCC is lower than that of low-grade ccRCC. Both high-grade ccRCC and type 2 pRCC often had prominent necrosis and some imaging findings overlapped. When high-grade ccRCC and type 2 pRCC progress to an advanced stage where surgery is impossible to remove, the two tumors are treated with different drugs [21]. As a result, differentiating between high-grade ccRCC and type 2 pRCC is clinically significant. Our research found that a CT-based radiomics nomogram integrating clinical variables and radiomics signatures had a high predictive value in diagnosing high-grade ccRCC and type 2 pRCC, with AUC values of 0.974 and 0.952 in the training and testing groups, respectively.

Various clinical and imaging features help distinguish high-grade ccRCC from type 2 pRCC [15, 22, 23]. Several clinical characteristics were included in our analysis, although most did not differ statistically between high-grade ccRCC and type 2 pRCC. TEV1, TEV2, REV1, and REV2 were statistically substantially different in identifying these two tumors (*p* < 0.001), confirming earlier research findings. According to Zhang et al. [22] and Liang et al. [15], ccRCC was more enhanced than pRCC because of the high degree of malignancy and the abundance of tumor neovascularization. In our study, necrosis, boundary, lymph node metastasis, and renal vein invasion were not statistically different between the two tumors. Delahunt et al. [4] and Waldert et al. [24] showed that type 2 pRCC was more malignant than ccRCC. This is most likely because they did not classify ccRCC. Low-grade ccRCC is less aggressive than high-grade ccRCC, and its occurrence is much higher than that of high-grade ccRCC.

Radiomics is an emerging research modality for studies of kidney tumors [11, 25, 26]. Erdim et al. [25] found that enhanced CT texture analysis based on machine learning could be used to identify benign and malignant tumors in the kidney with accuracy and an AUC value of 91.7% and 0.916, respectively. Wang et al. [11] studied 77 patients with RCC who underwent routine MRI preoperatively. These patients had 32 cases of ccRCC, 23 cases of pRCC, and 22 cases of chRCC, and a total of 39 radiomics features were extracted from the MRI images. ROC curves were created to demonstrate the diagnostic efficacy, and the results showed AUC values ranging from 0.631 to 0.951 for distinguishing ccRCC from chRCC; 0.688–0.955 for differentiating pRCC from chRCC; and 0.747–0.890 for differentiating ccRCC from pRCC. Nie et al. [27] selected 99 patients for a preoperative CT examination and separated them into a training cohort (*n* = 80) and a testing cohort (*n* = 19) to create a radiomics nomogram for discriminating renal angiomyolipoma with minimal fat from homogeneous ccRCC. A total of 14 useful features that were extracted from CMP and NP were used to build a radiomics nomogram. The training cohort (AUC, 0.896; 95% CI, 0.810–0.983) and the testing cohort (AUC, 0.949; 95% CI, 0.856–1.000) demonstrated the radiomics nomogram's good discriminatory efficacy. Its discriminating power was greater than that of the radiomics signature and the clinical factors model.

The nomogram, which combines several risk factors, is a simple and effective statistical prediction tool that is commonly used to evaluate medicine prognosis and outcomes [28]. Huang et al. [29] used a nomogram that incorporated clinical parameters with a radiomics signature to predict disease-free survival in non-small lung cancer. The diagnostic efficiency of the nomogram outperformed that of clinical variables alone. Our study attempts to develop a nomogram based on REV1 and Rad-score with AUC values of 0.974 and 0.952 in the training and testing groups to predict the likelihood of high-grade ccRCC. The AUC values for the model developed solely on clinical features in the training and testing groups were 0.921 and 0.872, respectively. The strong diagnostic performance of the nomogram over clinical characteristics alone would imply that the Rad-score has higher diagnostic utility in separating high-grade ccRCC from type 2 pRCC.

Comparing our analysis to earlier radiomics research reveals several distinctions and advancements. First, because high-grade ccRCC and type 2 pRCC typically have many overlapping radiological appearances in CT images, our study focused on separating the two. Second, four of the eight radiomics features collected from the three scanning phases originated from the CMP images, suggesting that the CMP images are more effective at distinguishing high-grade ccRCC from type 2 pRCC than the other two phases. Third, by combining clinical variables with radiomics features, this study constructed the radiomics nomogram, allowing for a more thorough assessment of tumor characteristics and more dependable outcomes. Finally, most previous studies perform their texture analysis of tumors in one dimension. In contrast, we analyzed the tumor using all tumor dimensions and gathered additional characteristics. In comparison to earlier studies that had only a few dozen acquired features, we obtained over 1,000 extracted from the 3D analysis. In addition, our sample was derived from numerous centers, making it the most considerable sample size for radiomics-based identification of high-grade ccRCC and type 2 pRCC.

Our study is not without limitations. First, the retrospective nature of our study implied that bias might be introduced in the sample selection and overestimate of diagnostic accuracy might occur, so external validation will be included in future studies. Second, for tumor analysis, we exclusively extracted radiomic features from precontrast phase, CMP, and NP imaging. Further features may be taken from CT four-phase images in the future to gain more radiomics information about the tumor. Third, the images in our research were acquired from a range of CT scanners manufactured by different companies. Although images have been standardized before feature extraction, the potential for error data consistency still exists. Fourth, manual 3D ROI segmentation is time-consuming and complex, especially for tumors with enormous size and/or indistinct borders. Further study should be directed towards establishing a more reliable and reproducible automatic segmentation method for renal cancers.

## 5. Conclusion

For a CT-based radiomics nomogram comprising the precontrast phase, CMP, and NP images, our study concludes that combining clinical considerations with radiomics characteristics is crucial. Our radiomics nomogram has good diagnostic performance and can preoperatively discriminate between type 2 pRCC and high-grade ccRCC. As a new research method, radiomics nomograms require extensive validation before they can be used clinically.

## Figures and Tables

**Table 1:** A CT scan parameters.

Manufacturer	Siemens	General Electric	Philips
Scanner model	Sensation 64	Discovery 750	Brilliance
Sequence	Axial	Axial	Axial
Gantry rotation time (s)	0.5	0.5	0.5
Tube voltage (kV)	120	120	120
Tube current (mA)	200	250–400	180–450
Detector collimation (mm)	64 × 0.6	64 × 0.625	64 × 0.625
Matrix	512 × 512	512 × 512	512 × 512
Pitch	1.0	1.375	1.0
Slice thickness (mm)	5	5	5
Corticomedullary phase (s)	30	30	30
Nephrographic phase (s)	80	80	80
Excretory phase (s)	180	180	180

s, second; kV, kilovolt; mA, milliampere; mm, millimeter.

**Table 2:** Clinical factors in the training cohort and testing cohort.

Clinical factors	*Training cohort (n* *=* *98)*		*p*	*Testing cohort (n* *=* *44)*		*p*
	ccRCC	pRCC		ccRCC	pRCC	
*Gender*			0.628			0.296
Male	39 (80%)	37 (76%)		15 (68%)	18 (82%)	
Female	10 (20%)	12 (24%)		7 (32%)	4 (18%)	
Age (years)	61 (54–65)	57 (51–67)	0.719	54 (50–66)	61 (55–71)	0.208
Maximum diameter (cm)	6.8 (4.7–8.9)	6.1 (4.5–7.5)	0.216	6.4 (4.1–8.3)	5.5 (3.4–7.5)	0.291
*Shape*			0.225			0.066
Round	23 (47%)	26 (53%)		6	12	
Not round	26 (53%)	23 (47%)		16	10	
*Location*			0.417			0.226
Left	25 (51%)	29 (59%)		8 (36%)	12 (55%)	
Right	24 (49%)	20 (41%)		14 (64%)	10 (45%)	
*Boundary*			0.065			0.033
Clear	16 (33%)	25 (51%)		6 (27%)	13 (59%)	
Blurred	33 (67%)	24 (49%)		16 (73%)	9 (41%)	
*Calcification*			0.258			0.240
Present	11 (22%)	16 (33%)		2 (9%)	6 (27%)	
Absent	38 (78%)	33 (67%)		20 (91%)	16 (73%)	
*Necrosis*			0.051			0.531
Present	38 (78%)	20 (41%)		15 (68%)	13 (59%)	
Absent	11 (22%)	29 (59%)		7 (32%)	9 (41%)	
*Renal vein invasion*			0.798			0.750
Present	10 (20%)	9 (18%)		8 (36%)	7 (32%)	
Absent	39 (80%)	40 (82%)		14 (64%)	15 (62%)	
*Lymph node metastasis*			0.647			0.757
Present	14 (29%)	12 (24%)		9 (41%)	8 (36%)	
Absent	35 (71%)	37 (76%)		13 (59%)	14 (64%)	
TEV1 (HU)	70 (48–84)	22 (17–34)	<0.001	59 (26–112)	15 (9–19)	<0.001
TEV2 (HU)	72 (58–83)	40 (26–57)	<0.001	81 (53–104)	27 (19–37)	<0.001
REV1	0.77 (0.58–0.91)	0.25 (0.17–0.38)	<0.001	0.64 (0.29–0.95)	0.17 (0.11–0.27)	<0.001
REV2	0.60 (0.50–0.73)	0.31 (0.22–0.49)	<0.001	0.65 (0.52–0.76)	0.26 (0.18–0.36)	<0.001

TEV, tumor enhancement value; REV, relative enhancement value; 1, corticomedullary phase; 2, nephrographic phase.

**Table 3:** Comparison of the diagnostic performance among the three models.

Model	*Training cohort*				*Testing cohort*			
	AUC (95% CI)	Accuracy%	Specificity%	Sensitivity%	AUC (95% CI)	Accuracy%	Specificity%	Sensitivity%
Clinical factor model	0.921	88.8	85.7	91.8	0.872	77.3	90.9	63.6
	(0.872, 0.966)				(0.78, 0.95)			
Radiomics signature	0.968	91.8	89.8	93.9	0.936	86.4	90.9	81.8
	(0.941, 0.990)				(0.876, 0.986)			
Clinical radiomics nomogram	0.974	93.9	98.0	89.8	0.952	88.6	100	77.3
	(0.944, 0.997)				(0.903, 0.992)			

*AUC*, the area under the curve; *CI*, confidence interval.

## Data Availability

The raw data supporting the conclusion of this article will be made available by the authors without undue reservation.

## Supplementary Materials

Supplementary Materials Supplementary Figure 1: details of the manual 3D segmentation of kidney tumors.
