# Supplementary material for: A Clinical Radiomics Nomogram Was Developed by Integrating Radiomics Signatures and Clinical Variables to Distinguish High-Grade ccRCC from Type 2 pRCC
Source: J Oncol. 2022 Aug 26;2022:6844349. doi: 10.1155/2022/6844349 (PMC9439906; doi:10.1155/2022/6844349)
Supplement: Supplementary Materials — Supplementary Figure 1: details of the manual 3D segmentation of kidney tumors. [file 6844349.f1.docx]

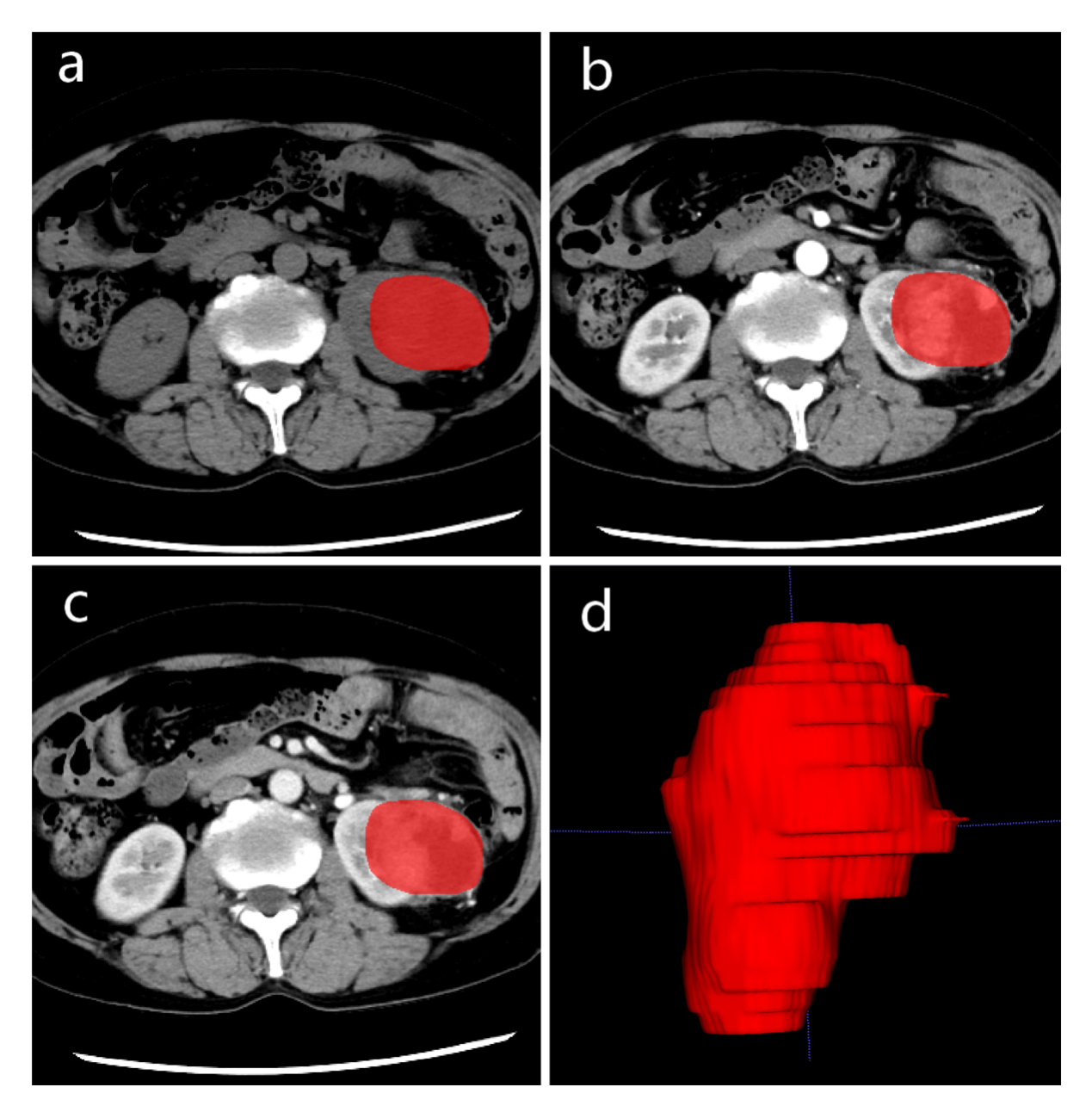


**Figure. S1** Manual three-dimensional (3D) of the tumour. a, b and c are the pre-contrast phase, the corticomedullary phase (CMP) and the nephrographic phase (NP), respectively. d is three-dimensional volumetric reconstruction.
